# Automatic Photo-Cross-Linking System for Robotic-Based In Situ Bioprinting

**DOI:** 10.1021/acsbiomaterials.3c00898

**Published:** 2023-10-12

**Authors:** Gabriele Maria Fortunato, Elisa Batoni, Ilenia Pasqua, Matteo Nicoletta, Giovanni Vozzi, Carmelo De Maria

**Affiliations:** Department of Information Engineering and Research Centre “E. Piaggio”, University of Pisa, 56122 Pisa, Italy

**Keywords:** photo-cross-linking, automatic device, robotic-based in situ bioprinting, gelatin methacryloyl

## Abstract

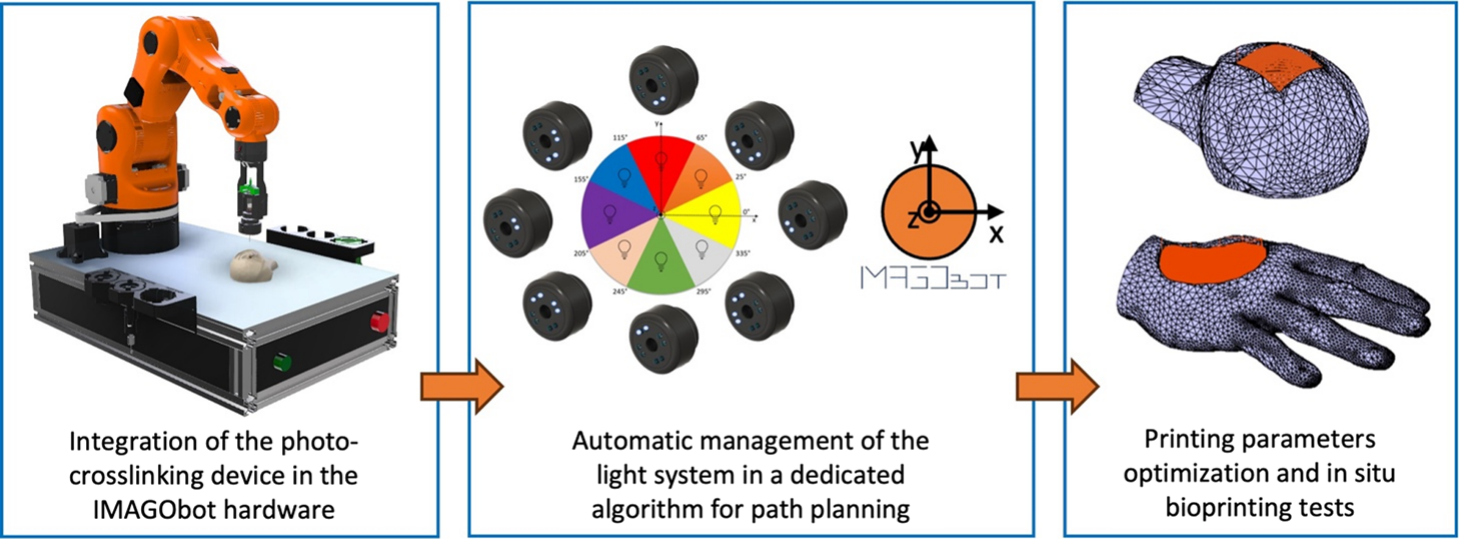

This work reports the design and validation of an innovative automatic photo-cross-linking device for robotic-based in situ bioprinting. Photo-cross-linking is the most promising polymerization technique when considering biomaterial deposition directly inside a physiological environment, typical of the in situ bioprinting approach. The photo-cross-linking device was designed for the IMAGObot platform, a 5-degree-of-freedom robot re-engineered for in situ bioprinting applications. The system consists of a syringe pump extrusion module equipped with eight light-emitting diodes (LEDs) with a 405 nm wavelength. The hardware and software of the robot were purposely designed to manage the LEDs switching on and off during printing. To minimize the light exposure of the needle, thus avoiding its clogging, only the LEDs opposite the printing direction are switched on to irradiate the newly deposited filament. Moreover, the LED system can be adjusted in height to modulate substrate exposure. Different scaffolds were bioprinted using a GelMA-based hydrogel, varying the printing speed and light distance from the bed, and were characterized in terms of swelling and mechanical properties, proving the robustness of the photo-cross-linking system in various configurations. The system was finally validated onto anthropomorphic phantoms (i.e., a human humerus head and a human hand with defects) featuring complex nonplanar surfaces. The designed system was successfully used to fill these anatomical defects, thus resulting in a promising solution for in situ bioprinting applications.

## 1 Introduction

In situ bioprinting is an emerging technology aiming at directly dispensing biomaterial inks/bioinks onto and into damaged anatomical sites in clinical environments following predefined bioprinting paths.^1−3^ There are two different approaches for in situ bioprinting: (i) the hand-held approach, which relies on portable devices moved by the surgeon’s hand to directly deposit materials on the defect site; (ii) the robotic approach, where three or more Degrees-of-Freedom (DoF) systems are used, enabling printing on sites with irregular shapes, ensuring less human interventions and higher accuracy and repeatability in movements. This novel technology gives the possibility to repair damaged tissues that, due to trauma or surgical excision, are usually characterized by curved surfaces and complex geometries.^4^

Compared to in vitro bioprinting, this technology presents several advantages such as the possibility to directly bioprint onto/into the irregular and complex defect site avoiding the need to remodel the scaffold due to an inaccurate fabrication, and the presence of a bioreactor no more necessary since the physiological environment presents biochemical and biophysical cues for creating functional tissues/organs.^1,5^ However, there are still some barriers to overcome before considering the technology robust for clinical applications: (i) printing onto/into non-planar surfaces may be challenging and not always possible; (ii) the bioprinting unit size might have dimensions not comparable to minimally invasive surgery techniques; and (iii) biomaterial inks/bioinks should be instantaneously cross-linked on the defect site while printing, to enhance the shape fidelity of the printed structures and so the final tissue formation.^3,5^

In the literature, two main different strategies have been described for cross-linking biomaterial inks/bioinks: (i) physical or reversible cross-linking, where polymer chains are linked together by secondary bonds (i.e., hydrogen bonding, hydrophobic interactions, ionic and electronic cross-linking); and (ii) chemical or irreversible cross-linking, where polymer chains are covalently bound (e.g., cross-linking with aldehyde, radical cross-linking, high energy irradiation cross-linking, photo-cross-linking, thermal cross-linking, enzymatic cross-linking).^6^ For in situ bioprinting applications, photo-cross-linking seems to be the most appropriate and promising method since it is rapid, durable, non-invasive, and easy to control through light intensity and time exposure.^7^ Photo-cross-linkable biomaterial inks/bioinks contain a photoinitiator, that is, a molecule or a combination of molecules, which generates reactive species (e.g., free radicals, cations, and anions) once the light source is absorbed, promoting the formation of chemical bonds.^8^ The photoinitiator wavelength must be accurately selected to avoid cell damage, optimizing its concentration to find a good compromise between cell viability and cross-linking time. Indeed, regarding printability, photoinitiators should have good solubility and compatibility with the polymer, good stability, low toxicity, and high reactivity, not influencing the final properties of the polymerized material.^9^ Among the light wavelengths, the UV range (320–365 nm) is the most used, even if it can potentially damage cells as well as operators. For this reason, visible light is preferred when it is possible. Gelatin methacryloyl (GelMA) is the most used photo-cross-linkable biomaterial ink/bioink for extrusion-based bioprinting (EBB). It can be cross-linked either with UV or with visible light sources, and it can be combined with other biomaterials (e.g., hyaluronic acid (HA), poly lactic acid (PLA), alginate, and poly(ethylene glycol) diacrylate (Alg/PEGDA)) to increase its initial viscosity. When blended with a water-soluble photoinitiator, GelMA methacrylate side groups establish covalent bonds forming a network of gelatin chains bound by poly methacryloyl ones. Other commonly used biomaterials, which are photocurable following the same mechanism of radical formation with the presence of the initiator, include PEGDA and methacrylate hyaluronic acid (M-HA).^8,10−13^ When processing these biomaterials with the EBB technique, the main challenge is to obtain a polymerization fast enough to maintain the printed shape due to the low initial viscosity. The most successful approach reported in the literature is “in printing” photopolymerization, where a light exposure system is used to irradiate the printing material through a photopermeable needle, obtaining a stable deposited filament.^14^ Both pre-cross-linking and post-cross-linking approaches gave non-acceptable results due to a heterogeneous and collapsed printed structure, respectively.^15^

When using photo-cross-linkable biomaterials inks/bioinks for in situ bioprinting, researchers have explored two different approaches: (i) photo-cross-linking post in situ bioprinting and (ii) photo-cross-linking during in situ bioprinting.

Considering the first approach, an example is reported by Di Bella et al.^16^ who used a hand-held device, featured with two separate cartridges, to repair a chondral defect of a large animal ovine model in a preclinical setting. Specifically, cartridges were loaded with two different bioinks, i.e., hyaluronic acid methacrylate (HAMA) and GelMA with a photoinitiator and HAMA-GelMA with mesenchymal stem cells (MSCs), deposited in a core/shell distribution inside the chondral defect. Then, the shell of the bioprinted strands was cured for 60 s with a UV light source (365 nm at an intensity of 10 mW/cm^2^) to provide mechanical support and protection to the embedded cells. Results demonstrated an overall enhancement of the regenerated cartilage macro/microarchitecture when compared to untreated control.^16^ In a similar study, Wang et al.^17^ developed a photo-cross-linkable hydrogel combining pectin methacrylate (PECMA) and GelMA for controlling hemorrhage bleeding in skin wounds. The hydrogel was injected into a bovine skin wound and then photo-cross-linked using a UV lamp for 120 s (365 nm at an intensity of 800 mW/cm^2^). In vitro results on porcine skin bleeding showed the rapid photo-cross-linking of the hydrogel and its ability to circumvent the bleeding and decrease the coagulation time by 39%.

Since in situ bioprinting is directly performed on a damaged site, the second approach, i.e., photo-cross-linking during in situ bioprinting, is preferred as the material is cross-linked during the deposition on the damaged site. For instance, O’Connel et al.^18^ used a hand-held device featuring a 365 nm UV source directed toward the extruder nozzle to photo-cross-link the fabricated 3D structures. In vitro experiments were performed with the GelMA/HAMA hydrogel seeded with human adipose staminal cells showing high viability (>97%) 1 week after bioprinting. Then, the authors redesigned the hand-held device to include a 405 nm light-emitted diode (LED) placed close to the tip of the nozzle.^19^ Bioprinting parameters and material formulations (based on GelMA, Gelatin type A, and HA (GelMA-Gel-HA)) were optimized for in situ photo-cross-linking during extrusion, enabling the possibility to draw 3D structures by hand including freestanding arches and miniature sculptures. To the best of our knowledge, there is only one example in the literature reporting a robotic-based approach featured with a photo-cross-linking unit. The in situ bioprinting platform of Li et al.^20^ consists of a small-scale robotic arm with a microsized dispenser valve, equipped with a double-light-source curing system to photo-cross-link a PEGDA biomaterial ink. The cross-linking unit employs two UV sources (365 nm with a total energy density of 940 mW/cm^2^) to homogeneously cross-link the material from two sides. Process parameters, such as nozzle velocity and frequency, droplet diameter, and curing time, are automatically controlled through a user interface. Initial trials were carried out printing 2D structures, and then a proof-of-feasibility was performed through in situ bioprinting on a curved surface to fill a 3D complex defect model.

Although these studies successfully reported different photo-cross-linking strategies during the in situ bioprinting procedure, the cross-linking is performed by light sources switched on during the whole process. This approach limits the possibility of tuning the exposure direction according to the predefined printing path, thus resulting in needle clogging during extrusion.

In this study, a photo-cross-linking unit was added to a previously developed platform to simultaneously photopolymerize hydrogels during the material extrusion controlling the exposure direction according to the printing path. The in situ bioprinting platform consists of a 5 DoF robotic arm, IMAGObot, developed in a previous study,^21^ equipped with different tools for surface scanning and reconstruction,^22^ and for pneumatic-based EBB, tested also for bioprinting on moving substrates.^23^ In this study, the EBB tool was integrated with a photo-cross-linking system based on 4 couples of LEDs activated according to the printing path direction in an automatic way. Experiments were performed using a GelMA biomaterial ink owing to its well-known biocompatible properties and extensive use in this field.^24−26^ Printing parameters (i.e., substrate-light distance, printing speed, and pressure) were optimized to fabricate 3D structures on irregular surfaces. The photo-cross-linking of the 3D-printed structures was evaluated and confirmed analyzing their swelling and mechanical properties.

## 2 Materials and Methods

### 2.1 Overview of the IMAGObot Platform

The experimental part was carried out using IMAGObot, a robotic biofabrication platform developed in a previous study.^21−23^ IMAGObot is a 5 DoF robotic arm designed starting from the open-source project MOVEO from BCN3D and re-engineered to be used for in situ bioprinting applications. IMAGObot is equipped with an electro-magnet as an end-effector allowing a fast and simple tool change to use different instruments during a single task.^22^ In this work, the photo-cross-linking device was integrated into the pneumatic-based EBB tool and used for bioprinting tests. The robot is controlled by the open-source software LinuxCNC,^27^ while the path planning is carried out using the previously developed Matlab R2023a application.^28^ This application allows to manage all the steps of a standardized in situ bioprinting procedure from the scanning of the anatomical defect to the path planning for tissue regeneration.^29^ The slicer allows the planning of both planar and non-planar printing paths,^30^ thus ensuring a proper material deposition even on complex surfaces.

IMAGObot LinuxCNC source code and the Matlab application have been released as an open-source project on the GitHub platform (https://github.com/CentroEPiaggio/IMAGObot) and are constantly updated.

### 2.2 Photo-Cross-Linking Device: Hardware

**Figure 1:**
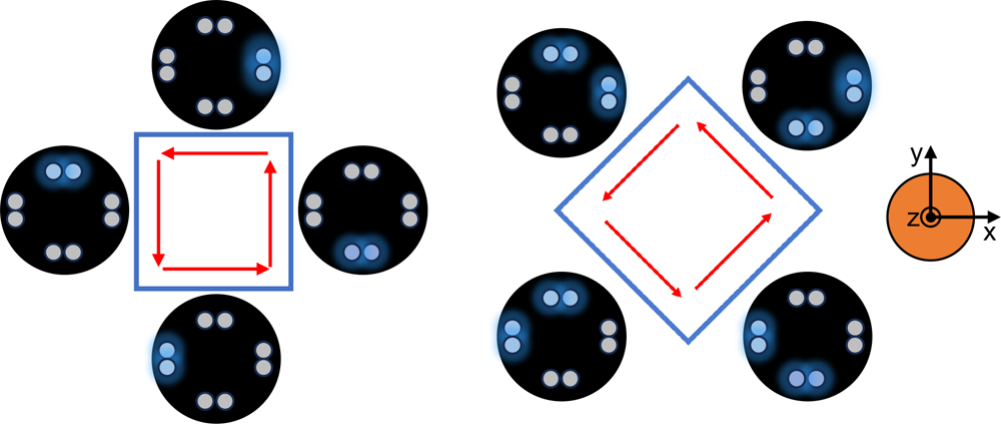
Working principle of the photo-cross-linking device. The 405 nm LED couple is switched on according to the opposite printing direction (blue: printing trajectory, red: printing direction, IMAGObot reference frame on the right). In diagonal trajectories, two couples of LEDs are switched on.

The working principle of the proposed device, integrated into the IMAGObot pneumatic extruder, is shown in Figure 1. The lighting system consists of 4 couples of LEDs (8 × 3 mm-diameter 405 nm LED - UV3TZ-405–30, Bivar Inc., USA) automatically switched on/off according to the printing direction. The aim is to minimize the exposure of the needle (placed in the middle) to avoid clogging. IMAGObot hardware and software were purposely re-engineered to enable automatic device management during the printing phase.

**Figure 2:**
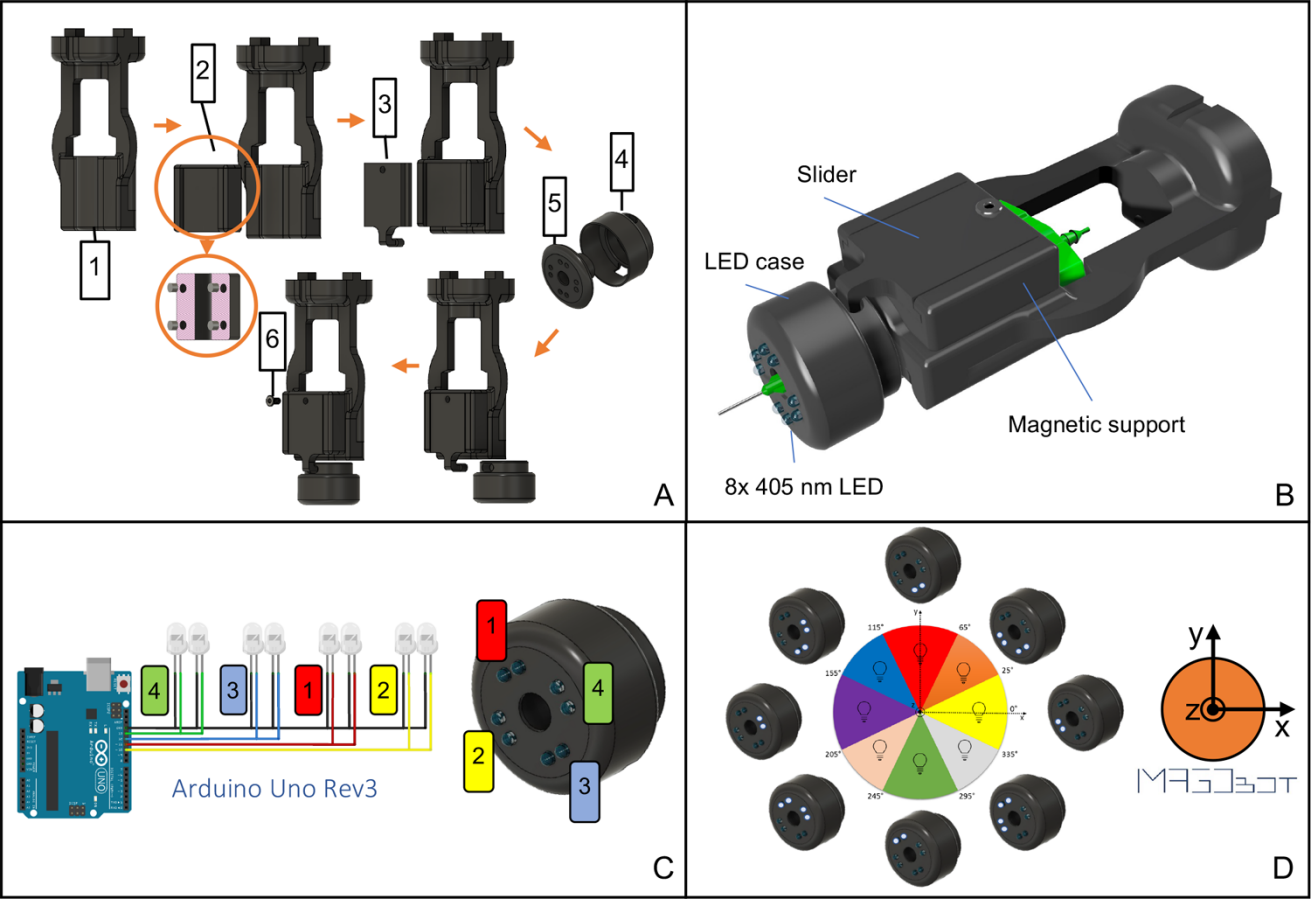
(A) Components and assembly diagram of the photo-cross-linking device: (1) syringe holder; (2) sliding guide with magnetic coupling; (3) slider; (4–5) LEDs housing; (6) M3 screw to adjust the slider position. (B) Assembled photo-cross-linking device. (C) Electrical connections: LEDs couples were connected to an Arduino Uno Rev 3 board. No resistors were used to obtain maximum light power (20–25 mA for each LED). (D) Schematic of the software working principle: the printing area was divided into eight angular sectors. According to the sector traveled during printing, the developed software enables the lighting of different LED couples.

A schematic of the device is shown in Figure 2A,B. It consists of a support attached to the printing needle that allows the housing of the lighting system. The LEDs are connected in parallel in pairs and at 90° to each other in the proximity of the syringe needle (at a distance of 10.6 mm). This configuration ensures the right exposure of the extruded material during printing and consequently promotes its instantaneous and complete photopolymerization. The components and the assembly diagram are shown in Figure 2A while the assembled device is shown in Figure 2B. All the components were 3D printed via Fused Deposition Modeling (FDM) using poly lactic acid (PLA). The presence of a slider allows the position of LEDs to be adjusted from a minimum of 1 mm to a maximum of 15 mm away from the printing substrate. The sliding movement has a 2-fold advantage since it allows both modulations of light exposure and printing on non-planar surfaces (compatible with a maximum depth of 15 mm). The wiring connection is shown in Figure 2C: an Arduino Uno Rev3 board was used to activate the proper combination of LEDs according to the printing direction. Since LEDs are connected in pairs to a digital pin (5 V output voltage, able to provide a maximum output current of 50 mA) and provide a maximum light power using a current of about 25 mA, no resistors were added in series with the LEDs.

### 2.3 Photo-Cross-Linking Device: Software

To enable automatic control of the LED pairs switching on/off, a Matlab script assigns the combination to be activated as a function of the planned print trajectories. The input of the algorithm is an ordered list of Cartesian coordinates representing the printing path. For each segment of the path, the projection on the XY plane is considered to identify the correct printing direction and thus the combination of LEDs to switch on/off. By using a polar coordinate system (Figure 2D), a versor is calculated for each segment of the trajectory, corresponding to the printing direction. By dividing the plane into 8 angular sectors of 45° each, an angle to the positive *x*-axis is then defined for each point. Knowing the angular sector, each segment of the trajectory is associated with a specific combination of LEDs, as shown in Figure 2D. Each of them is associated with a numeric code X (varying from 1 to 8) called by a custom M command (i.e., M102 PX) to automatically control the LED activation through the G-code, the standard code for piloting 3D printers.

### 2.4 Printing Parameters Optimization

**Figure 3:**
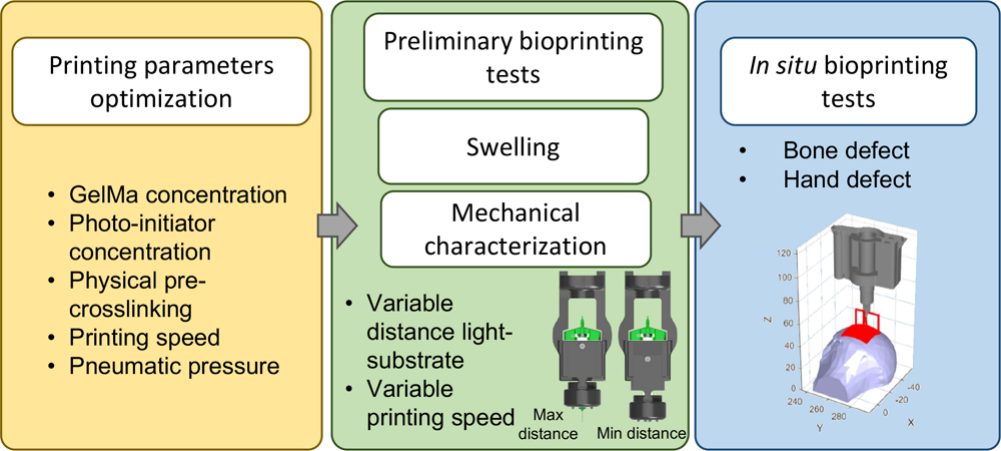
Overview of the experimental part for testing the photo-cross-linking device. First, the printing parameters were optimized for GelMA biomaterial ink analyzing the swelling and mechanical properties of the printed structures. Finally, an in situ bioprinting proof-of-feasibility was performed on two different anatomical bone and hand phantoms featuring a complex and irregular defect.

An overview of the experiments carried out for validating the photo-cross-linking device is reported in Figure 3. Preliminary tests were performed to optimize fabrication parameters. Gelatin methacryloyl (GelMa, Sigma-Aldrich, Italy) was used in different concentrations (5, 7.5, 10% w/v) for bioprinting tests and synthesized following the protocol described by Li et al.^31^ Lithium phenyl-2,4,6-trimethylbenzoylphosphinate (LAP, Sigma-Aldrich, Italy) was used as photoinitiator, testing two different concentrations (0.75–0.5% w/v). The GelMa + LAP biomaterial ink was physically pre-cross-linked at 4 °C before printing, testing different time intervals, i.e., 6, 9, and 12 min. Moreover, various combinations of printing speed (from 0.5 to 6 mm/s, step 0.25 mm/s up to 1 mm/s, and 1 mm/s up to 6 mm/s) and pneumatic pressure (from 0.3 to 1 bar, step 0.1 bar) were tested (see Table 2). During the parameter optimization phase, a monolayer grid (bounding box 20 × 20 × 0.2 mm) was printed using a 0.4 mm internal diameter needle. Printing fidelity was assessed by measuring the width of the deposited line by image analysis using the Fiji software on photos taken by a digital camera. As a control, a printing test was carried out, simultaneously switching on all the LEDs of the light exposure system.

The bioprinted structures were analyzed in terms of swelling and mechanical properties. Finally, in situ bioprinting tests were performed on anatomical phantoms (i.e., human humerus and hand) with an irregular complex defect using the previously optimized printing parameters.

### 2.5 Swelling and Mechanical Characterization

**Figure 4:**
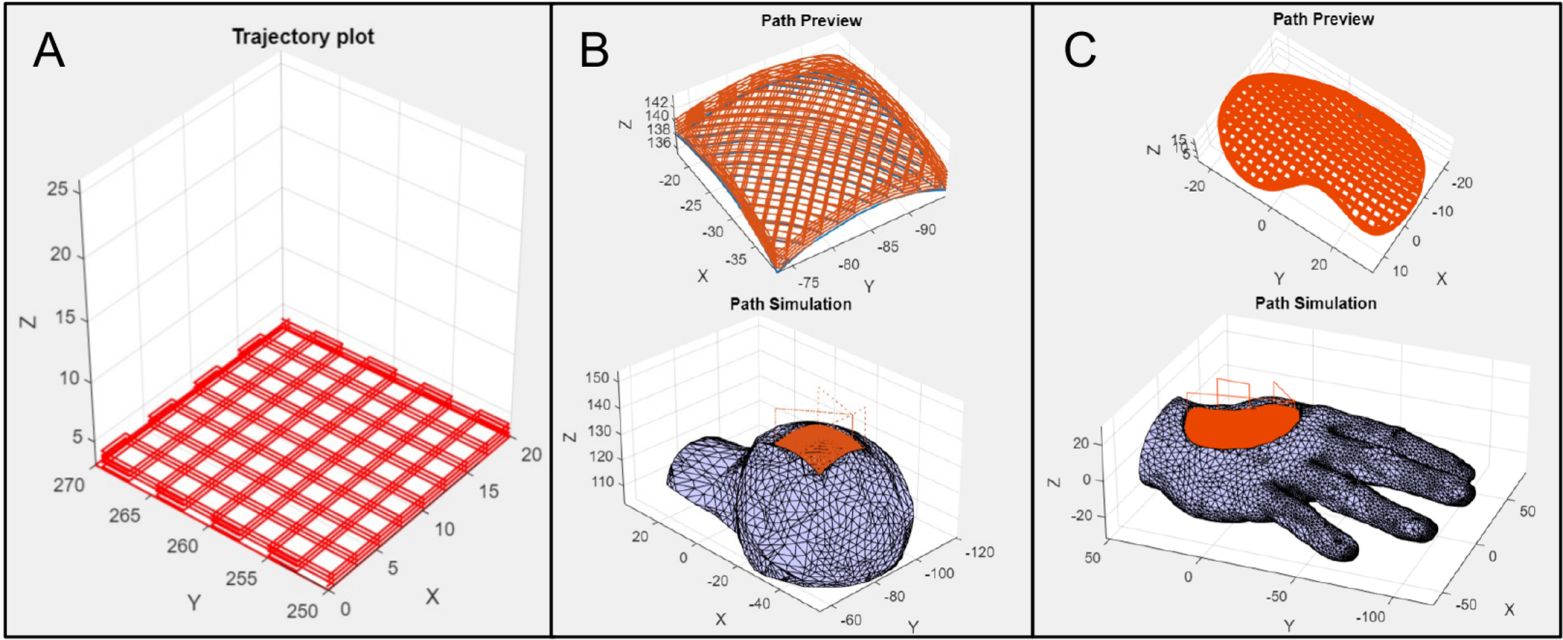
(A) Geometry printed for swelling and mechanical characterization: 6-layer 20 × 20 mm, layer thickness 0.2 mm. (B,C) Printing path planning on a human humerus head and a human hand featured with a complex nonplanar defect.

The ability of the photo-cross-linking device to correctly polymerize the deposited material during the printing phase was validated through swelling and mechanical characterization of GelMa + LAP structures fabricated with various combinations of optimized parameters. The 6-layer sample shown in Figure 4A (20 × 20 mm, layer thickness 0.2 mm, 50% infill percentage) was fabricated according to the parameters reported in Table 1. A 0.4 mm needle was used, and a red dye was added to the biomaterial ink for better visualization. For each combination of values, samples were prepared in triplicate. Swelling behavior was assessed up to 24 h in deionized water (DI) at 37 °C according to eq 1:
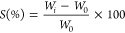
1where *W*_*i*_ represents the weight at the *i*-th time point (0.5, 1, 1.5, 2, 3, 4, and 24 h) and *W*_0_ is the initial weight.

**Table 1:** Printing Parameters for Swelling and Mechanical Characterization

	**printing speed** (mm/s)	**pneumatic pressure (bar)**
**min distance LEDs-substrate (1 mm)**	3	0.7
	4	0.8
	5	0.9
**max distance LEDs-substrate (15 mm)**	3	0.7
	4	0.8
	5	0.9

Mechanical characterization was obtained by performing a compression test (along the z direction, according to Figure 4A) with the universal uniaxial testing system Zwick/Roell ProLine Z005 equipped with a 100 N load cell. A 1% of the initial height was set as the strain rate, the sample was deformed up to 30% of the initial height, and the elastic modulus was determined for each specimen through a linear regression on the linear portion of the acquired stress–strain curves.

### 2.6 In Situ Bioprinting Tests

Two different in situ bioprinting tests were carried out on physiologically relevant phantoms. A human humerus head and a human hand, both with a defect, were used (Figure 4B,C). Starting from the .stl file of the missing portion (obtained as Boolean subtraction between healthy and defected anatomical regions), a non-planar printing path was planned, using the slicer previously developed.^28^ Non-planar layers were fabricated with a 50% infill percentage and a layer thickness of 0.2 mm. Structures with 8 and 15 layers were printed for the bone and the hand, respectively. The photo-cross-linking device was fixed at the maximum distance from the substrate while printing speed and pneumatic pressure were set at 3 mm/s and 0.7 bar for the bone and at 4 mm/s and 0.8 bar for the hand. Blue and red dyes were added to the GelMa + LAP hydrogel for the bone and the hand, respectively.

The bone with the repaired defect was placed in deionized water at 37 °C for 72 h immediately after printing to evaluate its stability in an aqueous environment (similar to the physiological one), thus demonstrating its photo-cross-linking. The possibility of handling the structure printed on the hand was also assessed to evaluate the polymerization of the deposited biomaterial ink.

### 2.7 Statistical Analysis

Collected data from swelling and mechanical characterization were analyzed with a two-way analysis of variance (ANOVA) using the software GraphPad Prism 8.0. Data are expressed as mean ± standard deviation.

## 3 Results and Discussion

### 3.1 Printing Parameters Optimization

**Figure 5:**
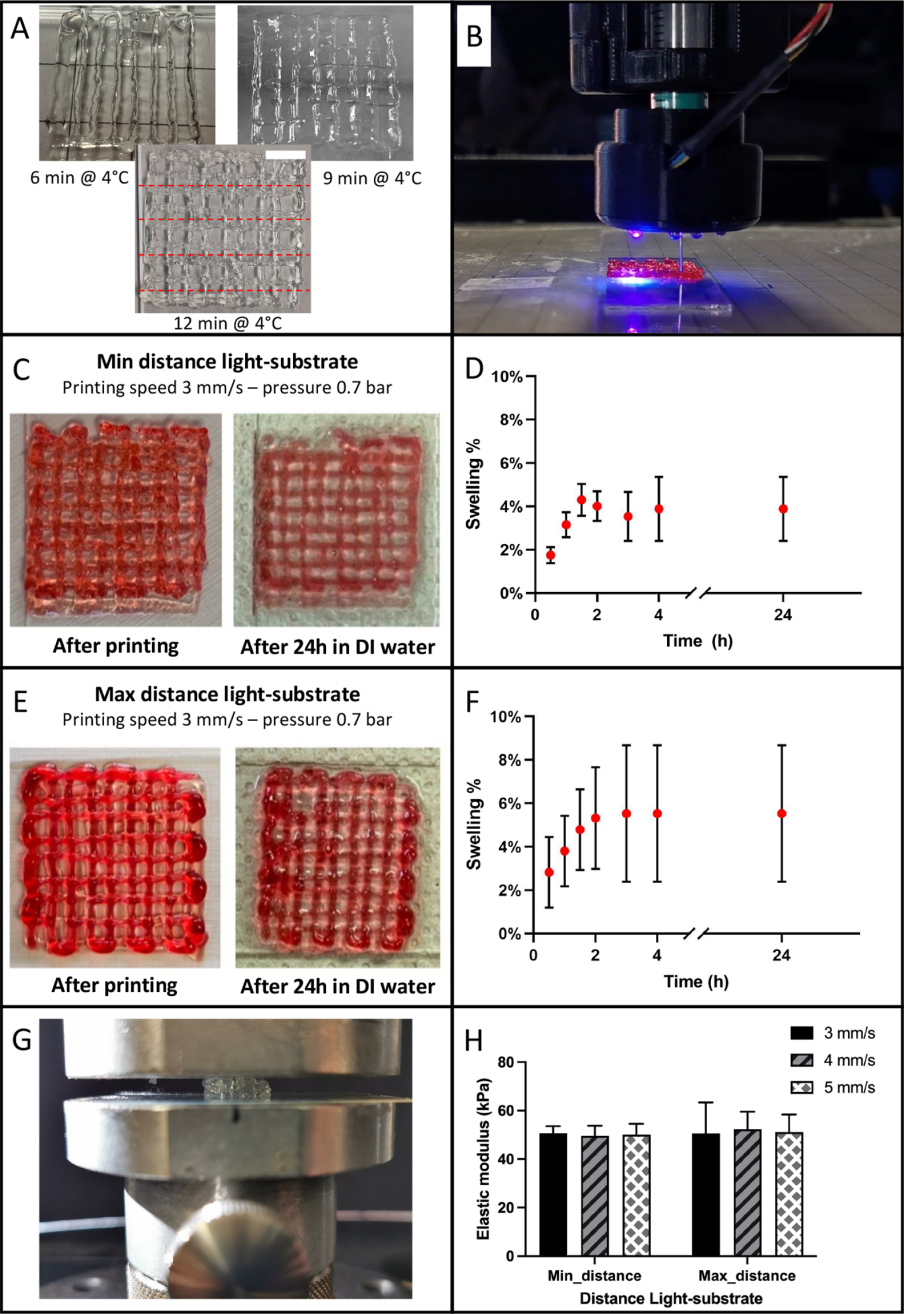
(A) Printing parameters optimization test: a physical pre-cross-linking of 12 min at 4 °C ensures a better printability of the GelMA + LAP hydrogel. Printing fidelity was assessed measuring the line width along dashed red lines as mean ± standard deviation. Scale bar = 500 μm. (B) Photo-cross-linking device during the printing phase of a six-layer structure for swelling and mechanical characterization. (C) Swelling test: sample after printing and after 24 h in deionized water (DI), min distance and min printing speed. (D) Swelling trend sample printed at min distance and min printing speed. (E) Swelling test: sample after printing and after 24 h in DI, max distance and min printing speed. (F) Swelling trend sample printed at max distance and min printing speed. (G) Compression test of the GelMa + LAP printed sample. (H) Elastic modulus of the samples printed varying the distance of the light system from the substrate and the printing speed.

Preliminary tests were performed to evaluate the best combination of bioprinting parameters. The photopolymerization of 7.5% w/v and 10% w/v GelMa was not achieved with both 0.5% w/v and 0.75% w/v LAP, thus, 5% w/v Gelma + 0.5% w/v LAP was used for all the subsequent tests. The inability to photopolymerize GelMa at the above two concentrations is due to the combination of process parameters used. Since there is no continuous exposure, the result is highly dependent on the printing speed and light-emitting power, which are not compatible with proper curing in these two cases. A different printability of this biomaterial ink was obtained by varying the time interval of the physical pre-cross-linking at 4 °C as shown in Figure 5A. Best performances were achieved after a pre-treatment of 12 min, obtaining a continuous and stable deposited filament. The pre-cross-linking phase ensured the proper printability of the material. Considering the maximum duration of the experiments (<30 min), no consistent modifications occurred on the viscosity of the GelMa-based biomaterial ink and on its processability. In the case of a longer printing time, a syringe temperature control system could be needed (e.g., based on a Peltier cell) to ensure a constant temperature of the biomaterial ink in the reservoir throughout the printing process. For each printing speed, a pneumatic pressure value was identified to obtain a line width similar to the printing needle’s internal diameter (0.4 mm), as reported in Table 2. Printing fidelity analysis was performed for each sample by measuring line width as mean ± standard deviation along dashed red lines, as shown in Figure 5A. Considering the sample printed at 0.3 mm/s and 0.7 bar, the line width was 0.52 ± 0.06 mm. The difference between this width and the needle's internal diameter could be due to both the die swell phenomenon and the collapse of the deposited strand under gravity. The latter, in the absence of a photopolymerization system, would be significantly higher, due to the low viscosity of the material. The use of the device proposed in this work reduces the collapse of the material by ensuring polymerization simultaneously with deposition, thus guaranteeing a better printing quality.

**Table 2:** Combinations of Printing Speed (*v*) and Pneumatic Pressure (*p*) To Obtain a Continuous Deposited GelMa + LAP Filament with a Line Width Similar to the Printing Needle Used (0.4 mm)

*v* [mm/s]	0.5	0.75	1	2	3	4	5	6
***p* [bar]**	0.3	0.4	0.5	0.6	0.7	0.8	0.9	1

The control test, carried out with all the LEDs switched on, gave no results since needle clogging occurred immediately after printing start due to excessive light exposure of the tip.

### 3.2 Swelling and Mechanical Characterization

As shown in Figure 5B, 6-layer structures were printed to evaluate swelling and mechanical behavior varying the distance of the light system from the substrate and the printing speed (see Supporting Information video 1.mp4). As an example, the swelling curves of two different samples (min distance–min speed and max distance–min speed) are reported in Figure 5 C–F. The swelling reaches a plateau after 2–3 h in deionized water (3–5% respect to the initial weight), and comparing this value with that at 24 h (as reported in Table 3) no statistically significant differences were found (*p*-value > 0.05 for both the row factor–printing speed– and the column factor–distance).

**Table 3:** Swelling Percentage after 24 h in Deionized Water at 37°C and Elastic Modulus for Samples Printed Varying the Distance between the Exposure System and the Substrate and the Printing Speed.^a^

	**printing speed** [mm/s]	**swelling % @24h**	**elastic modulus [kPa]**
**min distance LEDs-substrate**	3	3.61 ± 1.26	50.72 ± 2.85
	4	3.00 ± 0.66	49.56 ± 4.23
	5	3.56 ± 1.03	50.00 ± 4.55
**max distance LEDs-substrate**	3	5.53 ± 3.14	50.64 ± 12.71
	4	3.89 ± 1.48	52.32 ± 7.25
	5	4.80 ± 1.17	51.16 ± 7.20

a Data are reported as mean± standard deviation.

Elastic modulus was determined for each sample through a uniaxial compression test obtaining an average value of ∼50 kPa. Considering the mechanical characterization (data shown in Table 3), as for the swelling behavior, no statistically significant differences were highlighted taking into account both the exposure system-substrate distance and the printing speed (*p*-value > 0.05).

Swelling and mechanical properties are strongly related to the photo-cross-linking step, which can be quantified according to the cure depth *C*_d_, expressed as (eq 2):^32^
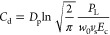
2where *D*_p_ [mm] is the penetration depth, *P*_L_ [mW] is the power of the light source, *w*_0_ [mm] is the diameter of the luminous spot on the substrate, *v*_s_ [mm/s] is the scanning speed, and *E*_c_ [mJ/mm^2^] is the critical energy. *D*_p_ and *E*_c_ are parameters typical of the resin and for 5% w/v GelMa + 0.5% w/v LAP are 0.61 mm and 0.48 mJ/mm^2^.^33^ Considering a LED pair, *P*_L_ = 60 mW (single LED emitting power 30 mW), thus . The spot diameter *w*_0_ varies from 10 mm (at min distance) to 20 mm (at max distance) while *v*_*s*_ matches with the printing speed (3–4–5 mm/s). To ensure a proper photopolymerization, *C*_d_ must be greater than the layer thickness. In the worst conditions (*w*_0_ = 20 mm, *v*_s_ = 5 mm/s) the cure depth is 0.34 mm, thus greater than the 0.2 mm layer thickness. Since the *C*_d_ value results higher at the minimum distance, the adhesion between printed layers will be stronger, justifying a lower variability in the swelling (Figure 5D vs Figure 5F) and mechanical properties (Figure 5H) at the shortest distance, compared to the largest.

### 3.3 In Situ Bioprinting Tests

In situ bioprinting tests were performed on anthropomorphic phantoms exploiting the ability of the developed device to print onto complex non-planar surfaces thanks to the adjustable position of the light system. Both bone and hand defects were correctly filled with the GelMa-LAP hydrogel obtaining a complete reconstruction of the damaged anatomical region (see Supporting Information video 2.mp4 and Supporting Information video 3.mp4) in about 6 and 25 min, respectively. The dimensions of the printed structures (particularly for the hand with a bounding box of 50 × 40 × 10 mm) and their completely non-planar geometry, confirm the robustness of the developed system, which ensures the photopolymerization of even physiologically relevant size constructs with complex shapes.

**Figure 6:**
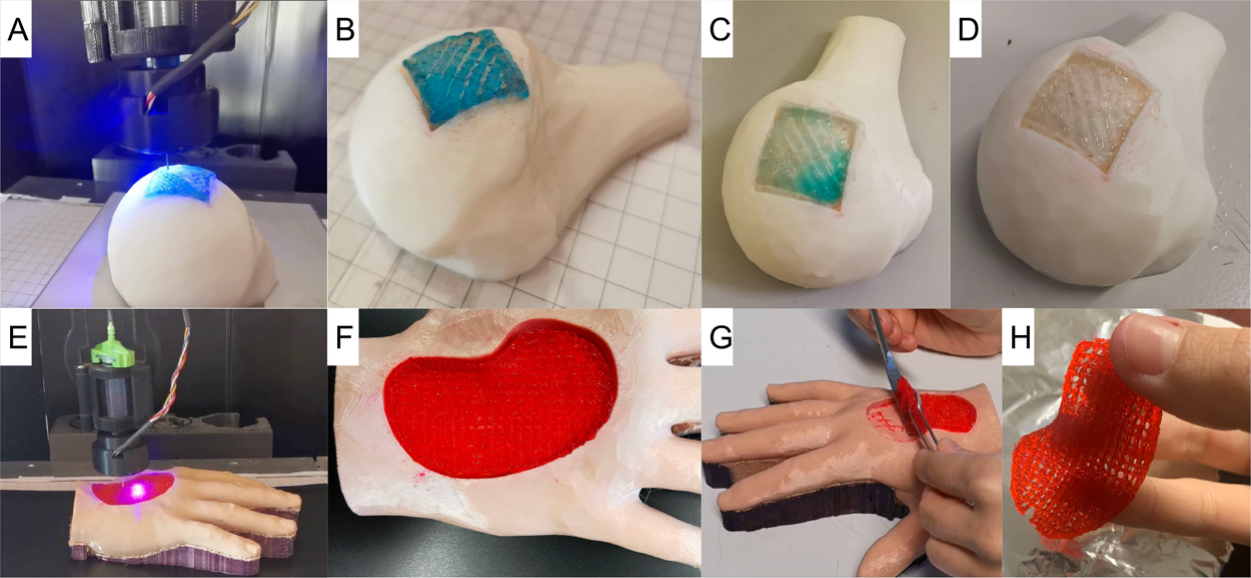
(A) Photo-cross-linking device during the printing step onto a human humerus head. (B) Bone sample with the filled defect immediately after printing. (C) Bone sample with the filled defect after 24 h in DI. (D) Bone sample with the filled defect after 72 h in DI (diffusion of the blue dye in DI is visible). (E) Photo-cross-linking device during the printing step onto a human hand. (F) Hand sample with the filled defect immediately after printing. (G,H) The stability of the printed construct enables high handling of the structure, which can also be easily removed from the printing site.

The stability of the printed construct on the first phantom (Figure 6A) was verified by placing the bone with the filled defect in deionized water at 37 °C immediately after printing (Figure 6B). Neglecting the blue dye that dissolved in water, the printed structure was not altered in the aqueous environment after both 24 and 72 h (Figure 6C–D). Qualitatively acceptable results were also obtained with the in situ bioprinting test on the human hand (Figure 6E,F). In this case, the printed structure proved to be correctly polymerized, as it was highly handleable after its removal from the printing site (Figure 6G,H).

Considering other works in the literature,^16−20^ the photo-cross-linking approach presented in our work enables for the first time the automatic control of the switching on/off of the light system according to the printing direction. Thanks to this innovative solution, the deposited material can be correctly polymerized also varying the exposure device configuration (i.e., distance from the substrate, printing speed) without any problems of needle clogging as may occur with other UV light systems. Moreover, the proposed system validated in this work for a GelMa-based biomaterial ink could potentially be used with other photocurable materials both optimizing the concentration of the material and selecting the proper type of photoinitiator and LED wavelength.

All the steps of the implemented algorithm to control the LED activation according to the planned printing path were included in the previously developed Matlab application. A standalone Matlab application with the implemented graphical interface has been released on the GitHub repository of the IMAGObot platform (https://github.com/CentroEPiaggio/IMAGObot).

## 4 Conclusions

In this work, an automatic photo-cross-linking device for robotic-based in situ bioprinting was designed and validated for different combinations of fabrication parameters. A dedicated software was also developed allowing the automatic control of the light system. According to the printing direction, different combinations of LEDs are switched on/off to polymerize the deposited biomaterial ink without any risk of needle clogging. The versatility of the developed system also allows for printing onto non-planar substrates, resulting very promising for in situ bioprinting applications where anatomical defects exhibit complex surfaces. The device was integrated into the previously developed IMAGObot system and successfully used for the reconstruction of two different anatomical defects. Moreover, the light control algorithm was integrated into the path planning software, thus allowing easy use even by non-expert users.

## Supporting Information Available

Supplementary video 1:3D printing of the 6-layer structure for swelling and mechanical characterization using the photo-cross-linking device (MP4)
Supplementary video 2: in situ bioprinting test using the photo-cross-linking device on the human humerus bone phantom (MP4)
Supplementary video 3: in situ bioprinting test using the photo-cross-linking device on the human hand phantom (MP4)
